# Supplementary material for: Risk factors and genotype distribution of hepatitis C virus in Georgia: A nationwide population-based survey
Source: PLoS One. 2022 Jan 21;17(1):e0262935. doi: 10.1371/journal.pone.0262935 (PMC8782338; doi:10.1371/journal.pone.0262935)
Supplement: S1 Appendix — (DOCX) [file pone.0262935.s001.docx]

Appendix for the manuscript titled “Risk factors and genotype distribution of hepatitis C virus in Georgia: a nationwide population-based survey”

**Authors**: Davit Baliashvili*^1,2^, Francisco Averhoff^3,^ Ana Kasradze^1^, Stephanie J. Salyer^4^, Giorgi Kuchukhidze^1^, Amiran Gamkrelidze^1^, Paata Imnadze^1^, Maia Alkhazashvili^1^, Gvantsa Chanturia^1^, Nazibrola Chitadze^1^, Roena Sukhiashvili^1^, Curtis Blanton^4^, Jan Drobeniuc^3^, Juliette Morgan^4,5,^, Liesl M. Hagan^3^

**S1 figure. Percentages of self-reported risk factors among anti-HCV-positive males, stratified by age.**

**
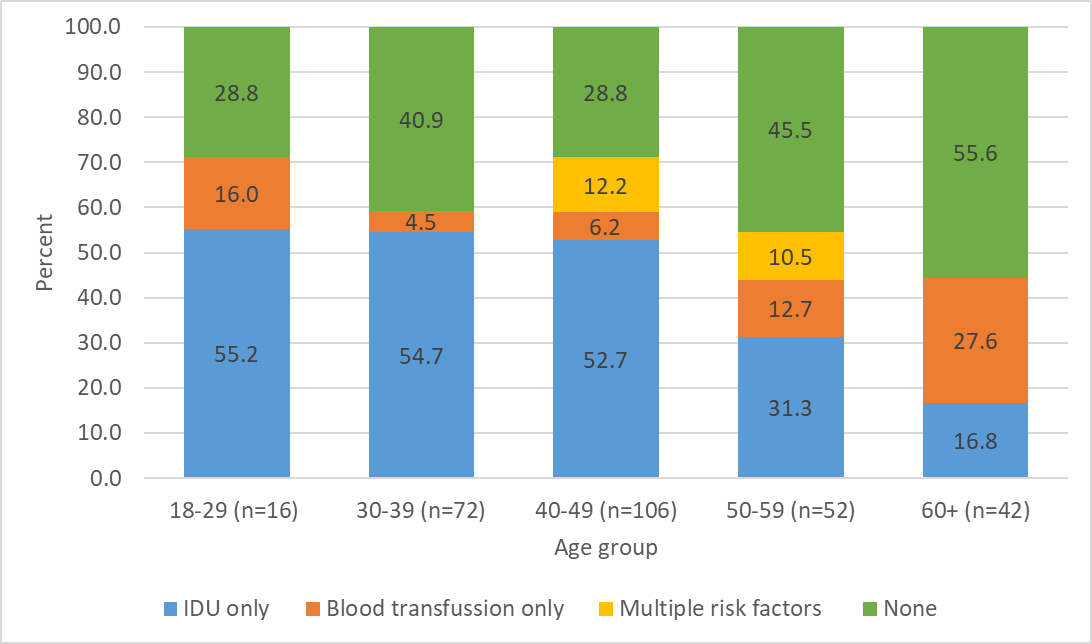
**

*Note*: The horizontal axis provides information about the age groups and number of participants in each of the age groups. The data labels on each graph shows the percentage of the participants in respective age group that reported the given risk factor.

*Abbreviations:* HCV=hepatitis C virus, IDU=Injection drug use.

**S2 figure. Self-reported risk factors among anti-HCV-positive females, stratified by age.
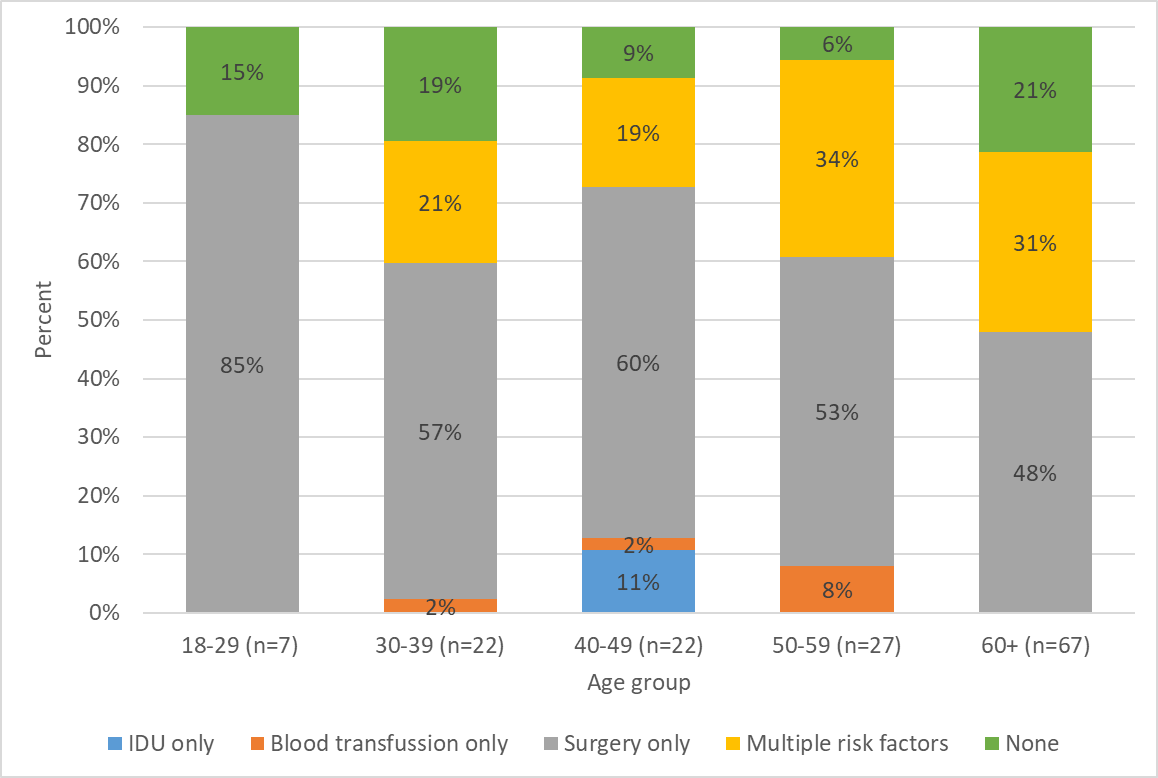
**

*Note*: The horizontal axis provides information about the age groups and number of participants in each of the age groups. The data labels on each graph shows the percentage of the participants in respective age group that reported the given risk factor.

*Abbreviations:* HCV=hepatitis C virus, IDU=Injection drug use.

**S3 figure. Receiver operating curves of HCV predictive models, stratified by sex.**

**
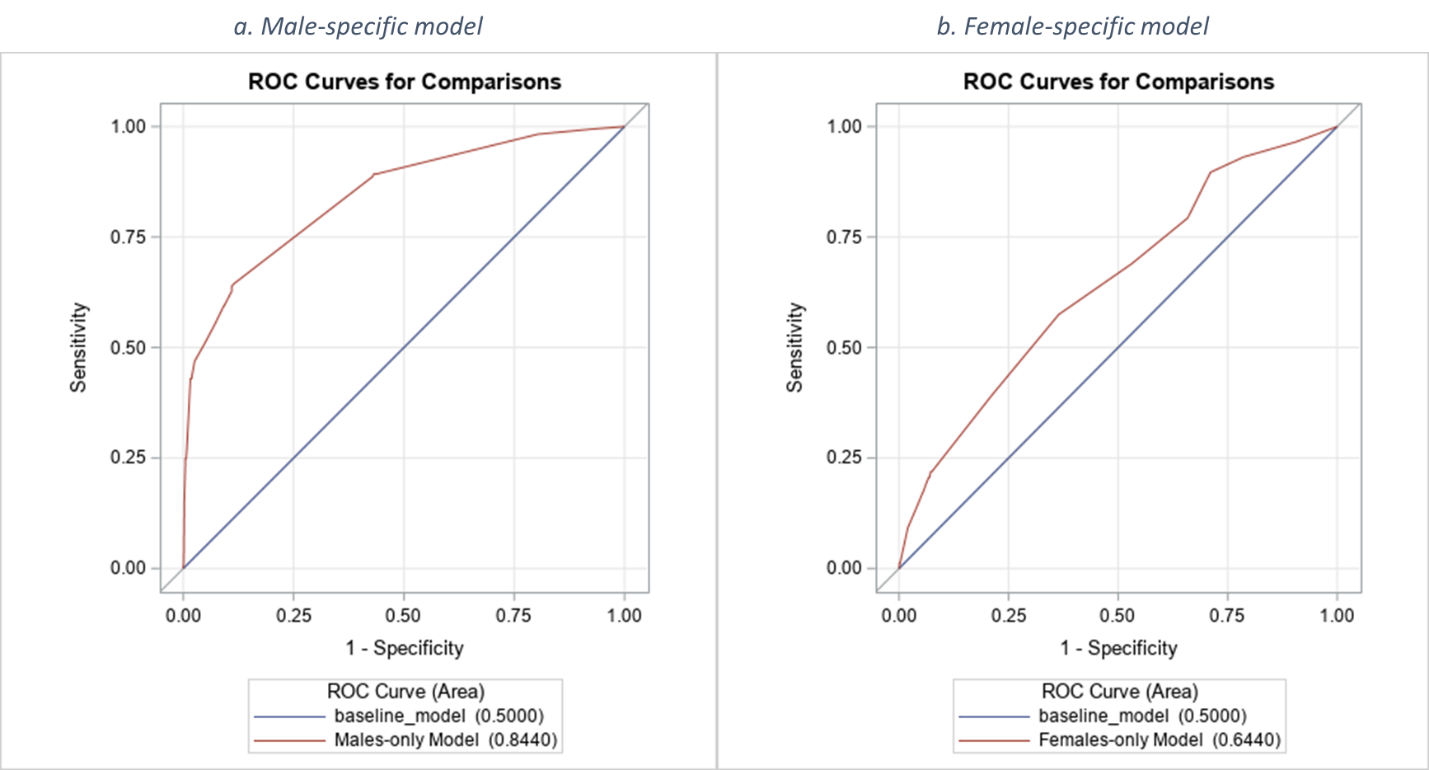
**

*Note:* The male-specific model included the following predictors: history of blood transfusion, history of injection drug use, history of incarceration, urban residency, and age (dichotomized at 30 years). The female-specific model included the following predictors: history of blood transfusion, history of surgery, history of dialysis, urban residency, and age (dichotomized at 50 years).

*Abbreviations:* HCV=hepatitis C virus, ROC=receiver operating curve.
